# *Curcuma longa* L. and Curcumin in Veterinary Medicine and Animal Production: Phytochemistry, Biological Mechanisms and Practical Applications

**DOI:** 10.3390/plants15111604

**Published:** 2026-05-23

**Authors:** Maria-Larisa Ardelean (Rusu), Florin Muselin, Alexandru Octavian Doma, Bogdan Florea, Romeo Teodor Cristina, Eugenia Dumitrescu

**Affiliations:** 1 Department of Pharmacology and Pharmacy, University of Life Sciences “King Mihai I” from Timișoara, Calea Aradului 119, 300645 Timișoara, Romania; maria-larisa.ardelean.fmv@usvt.ro (M.-L.A.); eugeniadumitrescu@usvt.ro (E.D.); 2 Doctoral School “Veterinary Medicine”, University of Life Sciences “King Mihai I” from Timişoara, Calea Aradului 119, 300645 Timişoara, Romania; bogdan-alexandru.florea.fmv@usvt.ro; 3 Department of Toxicology, University of Life Sciences “King Mihai I” from Timișoara, Calea Aradului 119, 300645 Timișoara, Romania; florinmuselin@usvt.ro (F.M.); alexandru.doma@usvt.ro (A.O.D.); 4 Department of Internal Medicine, University of Life Sciences “King Mihai I” from Timișoara, Calea Aradului 119, 300645 Timișoara, Romania

**Keywords:** *Curcuma longa* L., phytotherapy, curcumin, veterinary applications, essential oil

## Abstract

Interest in phytotherapy and phytogenic additives in veterinary medicine and animal production has increased considerably, driven by the search for functional alternatives to extensive antimicrobial use and the growing emphasis on food safety. In this context, *Curcuma longa* L. and its main bioactive compound, curcumin, have attracted attention because of their antioxidant, anti-inflammatory, immunomodulatory and antimicrobial properties. This review synthesizes recent evidence on the use of *C. longa* and curcumin in veterinary medicine, with emphasis on the botanical and phytochemical basis of the plant, the main biological mechanisms involved, and reported applications in poultry, swine, ruminants, aquaculture, and companion animals. It further highlights that the interpretation of findings is strongly influenced by botanical identity, phytochemical variability, product type, standardization, dose and route of administration. Available evidence indicates promising effects on antioxidant status, intestinal health, productive performance and hepatic protection in selected experimental models. However, translation into practice remains constrained by the low oral bioavailability of curcumin, formulation heterogeneity and inconsistent reporting. Overall, *C. longa* represents a promising phytogenic resource, but robust veterinary recommendations require studies in target species, better characterized products and standardized experimental protocols for application.

## 1. Introduction

**Figure 1:**
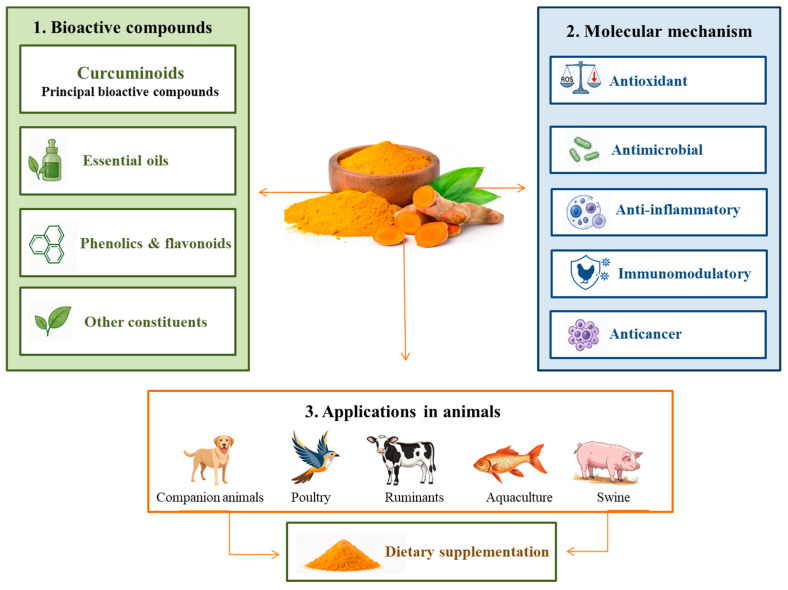
Overview of bioactive compounds, molecular mechanisms, and applications of *C. longa* in animal systems. Source: Author’s own elaboration using Microsoft PowerPoint.

The use of phytotherapy and phytogenic additives in veterinary medicine and animal production has increased significantly over the last decade, in the context of concerns regarding antimicrobial resistance and the need for sustainable alternatives to antibiotic use in intensive systems. In this framework, plant-derived compounds are of interest because they may act through multiple mechanisms, including antioxidant, anti-inflammatory, antimicrobial and immunomodulatory effects, contributing to the maintenance of animal health and performance. However, an essential aspect in the interpretation of results is the phytochemical composition and standardisation of preparations [1,2,3]. *Curcuma longa* L. (turmeric) is one of the most extensively studied plants with potential applications in veterinary medicine, mainly due to curcuminoids, particularly curcumin, and the volatile fraction of the rhizome, compounds associated with relevant effects in inflammation and oxidative stress [4]. The available studies indicate its use both in functional nutrition and as an adjuvant in different pathological conditions, including chronic disorders in companion animals [5]. However, the reported results are often inconsistent due to the significant variability in phytochemical composition; the type of product used, such as powder, standardised extract or advanced formulations; and differences in doses and administration protocols [3,6]. Although numerous studies have highlighted beneficial effects of curcumin on productive performance, intestinal health, antioxidant status and immune response in different animal species (Figure 1), the translation of these findings into practice remains limited by low oral bioavailability and the absence of standardised protocols. In addition, pharmacokinetic data suggest reduced systemic exposure, which raises questions regarding the actual mechanisms of action and the clinical relevance of the observed effects [7,8,9].

In this context, there is a clear need for a critical synthesis of the available data that takes into account differences between forms of administration, doses and animal species, as well as the methodological limitations of existing studies. Therefore, the aim of this review is to analyse the use of *C. longa* and curcumin in veterinary medicine, with emphasis on biological mechanisms, species-specific applications and critical aspects relevant to practice, including dose, formulation, safety and standardisation, while also highlighting existing gaps and future research directions.

### Literature Search and Selection Approach

This review is a narrative review that provides a critical synthesis of current evidence regarding the phytochemistry, biological mechanisms, and veterinary applications of *C. longa* and curcumin. A targeted literature search was conducted using PubMed/MEDLINE, Web of Science Core Collection, and Scopus to identify relevant studies published up to March 2026, including the latest available publications at the time of writing. Additional sources were identified through Google Scholar for supplementary citation tracking and the inclusion of recently published articles not yet fully indexed across databases. The final search was performed on 5 March 2026. The search strategy combined controlled vocabulary and free-text terms related to *C. longa*, curcumin, turmeric-derived products, and veterinary or animal production applications. Representative keywords included “*Curcuma longa*”, “curcumin”, “turmeric”, “curcuminoids”, “essential oil”, “turmerones”, “phytogenic feed additive”, “veterinary medicine”, “animal production”, “poultry”, “swine”, “ruminants”, “aquaculture”, “companion animals”, “antioxidant”, “anti-inflammatory”, “immunomodulatory”, “antimicrobial”, “bioavailability”, and “formulation”. Eligible studies included in vitro investigations, in vivo animal studies, and, where available, clinical or controlled studies relevant to veterinary medicine, animal nutrition, or animal health. Priority was given to studies reporting clearly defined biological, productive, or mechanistic outcomes, including antioxidant status, inflammatory markers, intestinal health, productive performance, hepatic protection, immune response, and pharmacokinetic or bioavailability-related parameters. Greater emphasis was placed on studies that provided adequate characterization of the plant material or product investigated, including botanical identification, plant part used, extraction method, curcuminoid profile, volatile fraction, formulation type, and route of administration. Particular attention was also given to studies in target animal species and papers discussing standardization, comparability of preparations, and limitations affecting translation into veterinary practice.

**Figure 2:**
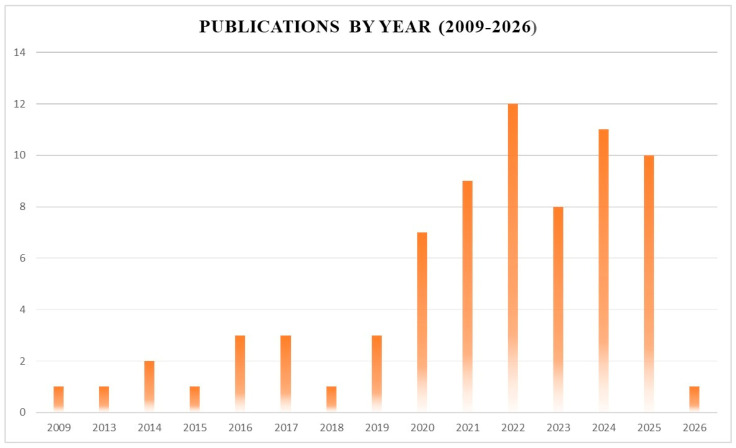
Annual distribution of publications related to *C. longa* and curcumin in animal studies (2009–2026). Source: Author’s own elaboration using Microsoft Excel.

**Figure 3:**
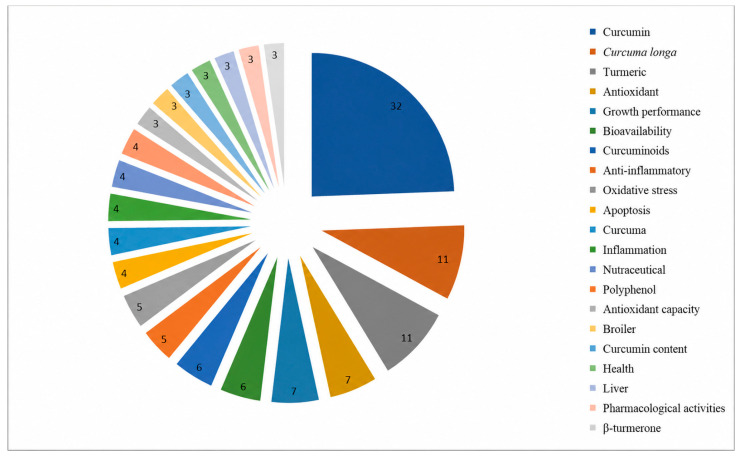
Frequency distribution of the keywords identified in the analysed literature. Source: Author’s own elaboration using Microsoft Excel.

In addition to the narrative synthesis described above, a complementary bibliometric analysis was conducted based on the 73 references included in this review, with the aim of further characterising research trends and thematic directions within the field. The analysed literature covers the period from 2009 to 2026, highlighting a progressive increase in scientific interest in *C. longa*, curcumin, and their applications in animal health and production. The annual distribution of publications is as follows: 2009: 1; 2013: 1; 2014: 2; 2015: 1; 2016: 3; 2017: 3; 2018: 1; 2019: 3; 2020: 7; 2021: 9; 2022: 12; 2023: 8; 2024: 11; 2025: 10; and 2026: 1 (Figure 2). One reference corresponds to a database source rather than a conventional scientific article. The highest number of publications was recorded in 2022, followed by 2024 and 2025, reflecting a marked intensification of research activity in recent years. The analysis of the references indicates that research on *C. longa* is distributed across a wide range of animal species, with a predominant focus on production animals. Poultry, particularly broiler chickens, represents the most frequently investigated category, followed by swine, ruminants, aquaculture species, and, to a lesser extent, companion animals and experimental models. This distribution highlights the increasing relevance of curcumin as a phytogenic feed additive in animal production systems. The bibliometric analysis included 73 scientific references, from which 372 keywords were identified, of which 240 were unique. To create the diagram, only keywords that appeared at least three times were selected in order to highlight the most relevant thematic directions in the analysed literature. Based on this criterion, the frequency diagram presented in Figure 3 was developed. The results show that the dominant term is “Curcumin”, with 32 occurrences, confirming the central role of curcumin in the included studies. The terms “*Curcuma longa*” and “Turmeric”, each with 11 occurrences, indicate the direct link between the bioactive compound and its plant source. The frequency of terms such as “Antioxidant”, “Growth performance”, “Bioavailability”, “Curcuminoids”, “Anti-inflammatory”, and “Oxidative stress” suggests that the main research directions are focused on the antioxidant and anti-inflammatory effects, bioavailability, and biological applications of curcumin. In addition, terms such as “Nutraceutical”, “Health”, “Liver”, and “Pharmacological activities” highlight the interest in the use of curcumin in health, nutrition, and pharmacological applications. Thus, the distribution of keywords shows that the analysed literature focuses mainly on the bioactive potential of curcumin and its relevance as an antioxidant, anti-inflammatory, and nutraceutical agent.

Overall, the bibliometric findings indicate a clear transition from general phytochemical and pharmacological research towards more applied studies in animal nutrition and veterinary medicine. This trend underscores the growing importance of *C. longa* as a multifunctional natural source with significant potential for improving performance, immune function, gut health, oxidative balance, and disease resistance in animals.

This review does not follow a systematic review protocol and does not include a formal risk-of-bias assessment or meta-analysis. Instead, it aims to provide a structured and critical narrative synthesis of the available evidence, with emphasis on the distinction between rhizome-derived preparations, extracts, essential oil fractions, and pure curcumin, as well as on the implications of formulation heterogeneity for interpretation and practical application.

## 2. Taxonomy, Botanical Description and Geographical Distribution

### 2.1. Taxonomy

**Figure 4:**
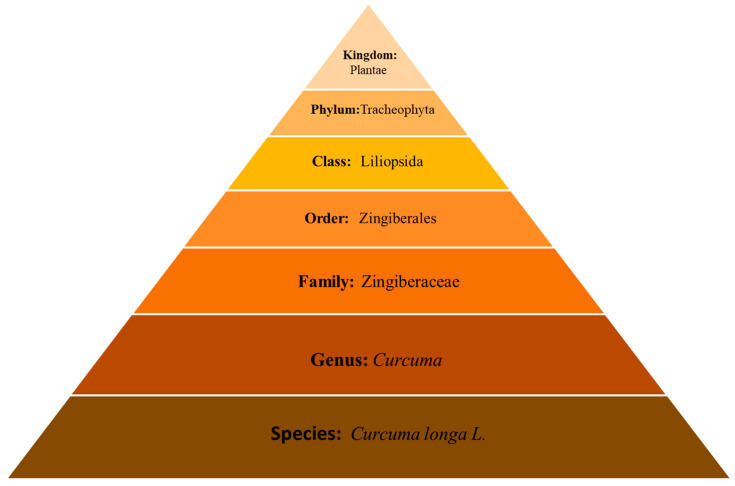
Taxonomy of *C. longa.* Source: Author’s own elaboration using Microsoft PowerPoint.

*C. longa* is taxonomically classified within the family Zingiberaceae and the genus *Curcuma* (Figure 4), a predominantly Asian group comprising numerous morphologically similar species, which may complicate the accurate identification of plant material and requires caution when assigning the species name [10].

Analyses based on chloroplast genomes suggest that phylogenetic relationships within the genus *Curcuma* can be effectively clarified through cpDNA (chloroplast DNA) comparisons at the genus level, providing a useful framework for delimitation and authentication [10]. In addition, a study integrating DNA barcoding and chemical fingerprinting across several *Curcuma* species highlighted variability among taxa and demonstrated the need for rigorous authentication of plant raw materials before interpreting phytochemical and biological results. This perspective is particularly relevant, as the absence of proper authentication may lead to the erroneous attribution of phytochemical profiles to *C. longa* when the analyzed material actually originates from other species within the genus [11]. Moreover, evidence of historical hybridization events reported within the genus *Curcuma* may further complicate taxonomic delimitation and reinforces the need to combine genetic markers with clear botanical descriptions and reproducible identification criteria [12]. Therefore, in the context of this review, the use of the full name *C. longa* and the explicit reporting of identification and authentication criteria represent essential conditions for the comparability of the studies synthesized below.

### 2.2. Botanical Description and Morphology

*C. longa* is a perennial rhizomatous herbaceous plant in which the underground rhizome functions as a perennation organ, supporting the regeneration of the aerial parts and the storage of resources [13]. From a genomic and cytogenetic perspective, the species is described as polyploid, and recent data for cultivated turmeric report a triploid chromosome number of 2*n* = 3× = 63. This status may have consequences for reproduction and for the uniformity of cultivated materials, which is relevant to the interpretation of comparisons between studies and the reproducibility of results [14]. The rhizome is the main economic organ of the plant and the principal source for most phytochemical investigations associated with turmeric, including the volatile fraction and essential oil. The literature also emphasizes that the essential oil profile depends on the characteristics of the raw material and on technological variables such as extraction method and processing, which makes it necessary to describe clearly the type of rhizome used and its condition in experimental reports [15].

In addition, a study on volatile constituents in several *Curcuma* species, including *C. longa*, showed that volatile profiling can support comparisons between batches and origins, thereby contributing to the characterization and control of variability among plant materials [16].

### 2.3. Reproduction, Cultivation and Variability

In agricultural practice, turmeric is propagated predominantly by vegetative means through rhizomes, a characteristic that may favor the maintenance of local lines but may also contribute to the persistence of regional differences depending on the planting material and production systems. In this context, a study on Indian germplasm highlighted variability among accessions both in rhizome traits and in curcuminoid content, indicating that *C. longa* may encompass a considerable range of phytochemical profiles and agronomic characteristics [17]. In addition, recent genomic data suggest that particular features of the genome and chromosomal architecture may be associated with biochemical differences, reinforcing the need for rigorous characterization of the plant material used in experimental and applied studies [14].

### 2.4. Geographical Distribution

**Figure 5:**
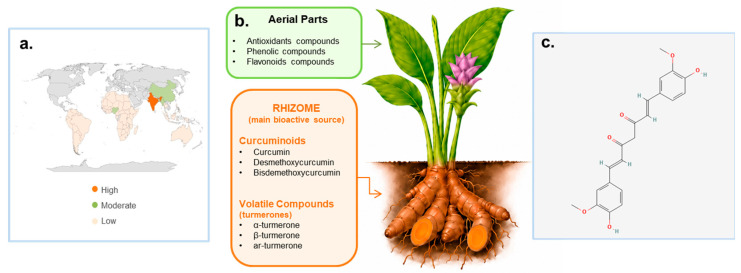
Overview of *C. longa*. (**a**) Global distribution of *C. longa*. Countries are classified according to the level of cultivation and production potential: high, moderate or low. (**b**) Botanical structure of *C. longa*, highlighting the rhizome as the main source of bioactive compounds, including curcuminoids such as curcumin, desmethoxycurcumin and bisdemethoxycurcumin, as well as volatile compounds, namely turmerones. (**c**) Chemical structure of curcumin. Adapted from PubChem [25]. Source: Author’s own elaboration using Microsoft PowerPoint.

*C. longa* is a rhizomatous species widely cultivated in tropical and subtropical regions worldwide because of its adaptation to warm and humid environments and its economic importance as a source of spice and bioactive compounds, including curcuminoids [18]. Membership of the family Zingiberaceae and its current predominantly agricultural distribution reflect the expansion of cultivation across many warm regions of the world, such as Asia, Africa and South America, where tropical and subtropical conditions favor rhizome development [19,20]. A major concentration of turmeric resources is found in South and Southeast Asia, as well as in cultivation-associated regions including India, China, Thailand, Singapore, the Philippines, Malaysia, Indonesia and Australia, suggesting a global distribution that is largely dependent on agricultural practices and trade. From a production perspective, India dominates global turmeric production, accounting for approximately 80% of the total, while other countries such as China, Myanmar, Nigeria and Bangladesh contribute smaller but still significant shares (Figure 5) [18]. Studies on germplasm indicate high genetic and phenotypic diversity among accessions cultivated in India, reflected in variation in rhizome traits and curcuminoid content, which confirms the role of this region as an important reservoir of genetic resources for cultivated turmeric. This variability among accessions, documented through agronomic and metabolic differences, suggests that geographical origin and planting material may influence the physicochemical characteristics of the raw material used in studies [17].

In addition, local agro climatic factors, such as minimum relative humidity, altitude, nitrogen content and soil pH, have been significantly associated with variation in curcumin content in rhizomes, indicating a link between the cultivation environment and phytochemical profiles [21]. In addition to the curcuminoid fraction, the essential oil composition of *C. longa* rhizomes also shows variability according to variety and cultivation conditions, with differences reported in the abundance of major volatile components among the analyzed materials. Studies on essential oils obtained from rhizomes cultivated in the northern United States, specifically in Alabama, identified dominant compounds such as α-turmerone, ar-turmerone, β-turmerone, 1,8-cineole, β-sesquiphellandrene, α-phellandrene and α-zingiberene, with proportions varying among varieties, suggesting chemical plasticity associated with genotype and agroclimatic context [19].

At the same time, it has been emphasized that the essential oil profile is also influenced by the characteristics of the raw material and the extraction method, which makes it necessary to report these parameters when comparing materials derived from different regions [15]. Overall, the available evidence indicates that the geographical distribution of *C. longa* reflects not only its cultivation range but also the interaction between genetic variability and environmental factors, which shape its phytochemical composition, including the essential oil profile, with direct implications for the comparative interpretation of biological results and the agricultural and biomedical applications of turmeric [19,21].

## 3. Phytochemical Composition of *C. longa* and Main Determinants of Chemical Variability

### 3.1. General Phytochemical Profile of the Rhizome

The rhizome of *C. longa* contains two major phytochemical fractions: a non-volatile fraction, mainly represented by curcuminoids, and a volatile fraction consisting of an essential oil rich in terpenoids, especially turmerone-type sesquiterpenes [15]. The recent literature highlights the importance of rhizome chemical characterization for the standardization of turmeric-derived products and explaining batch-to-batch variability [18]. It is important to relate phytochemical descriptions to the plant organ analysed, since most available data concern the rhizome, and its composition may differ from that of the aerial parts (Figure 5) [15]. In addition, metabolomics studies indicate that quality assessment may benefit from fingerprinting approaches and marker panels rather than relying only on a single compound [22,23].

### 3.2. Curcuminoids

Curcuminoids are the main markers used for the identification and quality control of *C. longa*. Curcumin is usually the predominant constituent, accounting for 60 to 75% of the curcuminoid fraction, followed by desmethoxycurcumin and bisdemethoxycurcumin (Figure 5) [24].

At the rhizome level, total curcuminoids may represent about 3 to 5% of the total mass, although their content depends on plant material and processing conditions [26]. Agro-ecological factors such as relative humidity, altitude, soil nitrogen and soil pH may significantly influence curcumin content, making provenance and cultivation conditions important when comparing reported values [21]. Genomic studies also provide a useful framework for understanding the biosynthesis and regulation of curcuminoids in *C. longa* [27]. Furthermore, DNA barcoding combined with HPLC (High-Performance Liquid Chromatography) fingerprinting has shown interspecific differences within the genus *Curcuma*, supporting the need for reliable botanical authentication in comparative phytochemical studies [11]. Some markers may still show limited discriminatory power between closely related taxa, which reinforces the need for careful species verification before interpretation [28].

### 3.3. Essential Oil

The essential oil of turmeric represents the volatile fraction of the rhizome and is commonly described as being rich in sesquiterpenes, particularly turmerone-type compounds [15]. Differences in the abundance of major constituents such as α-turmerone, β-turmerone and ar-turmerone have been reported among varieties cultivated under different conditions [19]. More broadly, the volatile profile of *Curcuma* species, including *C. longa*, may vary according to both genetic and environmental factors [16]. Therefore, comparisons of volatile profiles should take into account not only origin and variety but also the extraction procedure and operating conditions, since these can substantially affect the reported composition [15]. Recent genomic evidence also suggests that variation in terpenoid biosynthesis may be associated with structural features of the genome and terpene synthase-related genes [14]. Overall, the rhizome should be regarded as a complex phytochemical matrix combining volatile and non-volatile constituents [29].

### 3.4. Minor Metabolites, Mineral Elements and the Influence of the Cultivation System

Untargeted analytical approaches, including HPLC-MS/MS (High-performance liquid chromatography–tandem mass spectrometry) and molecular networking, have revealed additional metabolites in rhizome extracts beyond the classical curcuminoid profile, supporting the value of chemical fingerprinting for sample comparison [22]. In addition, the mineral composition of the rhizome may vary according to soil conditions and fertilisation practices [30]. The rhizosphere microbiome and plant growth-promoting microorganisms may also influence nutrient availability, plant physiology and the accumulation of secondary metabolites, including curcumin and related compounds [31].

### 3.5. Implications for Standardization and Comparability of Studies

The literature indicates that phytochemical variability in *C. longa* may result from genetic background, provenance, cultivation conditions, post-harvest processing and extraction methods [15]. For this reason, studies should clearly report the type of plant material analysed, the extraction system and the analytical method used, particularly for curcuminoid quantification [26]. Similarly, comparisons of the volatile fraction should be interpreted in relation to the essential oil isolation method and extraction conditions [15]. The integration of genomic tools with phytochemical data may improve understanding of the biological basis of chemical variability [27]. In parallel, data from DNA barcoding and HPLC fingerprinting show that chemical profiles differ among *Curcuma* taxa and samples, making botanical identity and chemical characterization essential for the interpretation of biological results [11].

## 4. Biological and Functional Mechanisms of Compounds from *C. longa*

**Figure 6:**
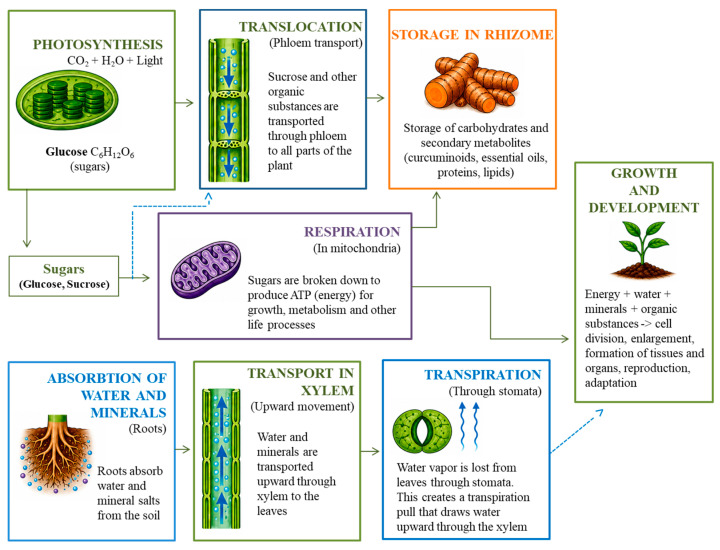
Schematic overview of the main physiological processes in *C. longa* associated with the synthesis, transport, and accumulation of bioactive compounds. Source: Author’s own elaboration using Microsoft PowerPoint.

To support the understanding of the biological and functional mechanisms of compounds derived from *C. longa*, a schematic overview of the key physiological processes involved in their synthesis, transport, and accumulation is presented in Figure 6.

### 4.1. Antioxidant Activity and Cytoprotective Response

Curcumin has been reported to modulate the cellular antioxidant response through activation of Keap1–Nrf2 (Keap1—Kelch-like ECH-associated protein 1; Nrf2—Nuclear factor erythroid 2–related factor 2) signaling and stimulation of the expression of genes dependent on the ARE element (antioxidant response) [32]. Activation of Nrf2 is associated with the induction of transcription of ARE-dependent cytoprotective genes, including HO-1 (heme oxygenase-1) and NQO1 (quinone oxidoreductase 1), supporting a transcriptionally regulated antioxidant mechanism. In addition, recent reviews describe antioxidant networks as being interconnected with other redox signaling nodes, and curcumin is discussed in this context as a multi-target agent capable of simultaneously influencing several cellular response pathways [33].

Curcumin is therefore discussed as a modulator of the Nrf2/ARE axis, a conserved mechanism of the cellular antioxidant response, which links rhizome-derived metabolites with adaptation to oxidative stress [32].

### 4.2. Anti-Inflammatory Activity

Species of the genus *Curcuma* may act on targets within signaling pathways, inhibit pro-inflammatory enzymes, and reduce the production of inflammatory cytokines and chemokines, thereby supporting the concept of an anti-inflammatory effect through the reduction of pro-inflammatory mediators and the modulation of signaling pathways. Curcumin is discussed as a modulator of inflammation and has been reported to suppress NF-κB (nuclear Factor kappa B) activation in certain models and reduce downstream inflammatory mediators, including COX-2 (cyclooxygenase) and iNOS (inducible Nitric Oxide Synthase) production [34]. Curcumin has also been reported to inhibit activation of the NLRP3 (the NOD-like receptor (NLR) family, the pyrin domain containing 3) inflammasome in a cell model based on THP-1 (monocytic cell line) cells differentiated into macrophages with PMA (phorbol 12-myristate 13-acetate). This effect was evidenced by reduced NLRP3 expression, decreased caspase-1 cleavage, and diminished IL-1β (interleukin-1 beta) maturation and secretion. The authors associated these effects with inhibition of TLR4 (toll-like receptor 4) and MyD88 (myeloid Differentiation Primary Response 88) signaling and reduced NF-κB activation. The involvement of the purinergic receptor P2X7R (purinergic 2 × 7 receptor) was supported by the finding that P2X7R silencing attenuated the inflammatory response and reduced phosphorylation of the NF-κB p65 subunit, together with a decrease in inflammasome markers, suggesting a role for P2X7R in amplifying inflammasome activation through NF-κB-dependent pathways [35].

### 4.3. Antimicrobial Activity

Curcumin has been described as having anti-infective properties, including antibacterial activity, and it has also been reported to act on multiple targets and mechanisms in experimental models. Within the same framework, effects on virulence-related processes and biofilm formation have also been reported, including interference with quorum sensing, which is particularly relevant for bacterial phenotypes that are difficult to control in infections [36]. The comparative interpretation of antimicrobial studies using curcumin should be made in relation to the form and formulation administered, since poor solubility, limited stability in aqueous media and low bioavailability may alter effective exposure and influence the reported outcomes [37,38].

### 4.4. Immunomodulation

Immunomodulatory effects have been reported for species of the genus *Curcuma* and their compounds, with *C. longa* being identified as the most extensively investigated species in the literature. These effects are discussed mainly in relation to changes in inflammatory mediators and cytokines, as well as the involvement of intracellular signaling pathways, including nodes such as NF-κB, in certain experimental models. At the same time, an important methodological limitation has been highlighted: many of the reported findings are derived from poorly characterized and non-standardized crude extracts, which makes the precise attribution of mechanisms to a specific metabolite more difficult [39].

### 4.5. Immunomodulation Contribution of the Volatile Fraction (Turmerones) and Distinction of Its Mechanisms from Those of Curcuminoids

It has been reported that the essential oil of turmeric rhizome contains α-turmerone, ar-turmerone and β-turmerone as its main constituents, and these compounds have been associated with biological effects in certain models. In this context, the available data suggest that the volatile fraction may contribute to bioactivity through mechanisms reported for turmerones and other essential oil constituents, which may only partially overlap with those attributed to curcumin [18]. In a study based on activity-guided isolation, the dichloromethane fraction showed the highest anti-inflammatory activity, and among the compounds subsequently isolated, β-turmerone was the most active [40]. When interpreting the applied sections, it is advisable that the type of product derived from *C. longa* be reported explicitly, for example, essential oil versus the non-volatile fraction or a curcuminoid-rich extract, because the chemical profile and bioactivity may depend on the fraction analyzed and the conditions of preparation, thereby reducing the risk of unjustified generalizations at the species level [15].

Table 1 summarizes the main biological and functional mechanisms associated with compounds from *C. longa*.

## 5. Veterinary Applications of *C. longa*

In veterinary medicine, *C. longa* and curcumin have been investigated mainly as phytogenic feed additives and supportive nutraceuticals, particularly in production animals and aquaculture. The available evidence suggests potential benefits in growth performance, antioxidant balance, intestinal health and resilience to inflammatory or toxic challenges. However, the strength of evidence varies among species, and the reported outcomes are influenced by formulation, dose and study design.

### 5.1. Poultry

In poultry production, curcumin has attracted considerable interest as a feed additive capable of supporting productive performance, intestinal function and oxidative balance. A recent meta-analysis including 28 studies reported increases in average daily gain and improvements in feed conversion in broiler chickens receiving dietary curcumin, together with improvements in antioxidant status and intestinal morphology. The doses used in experimental studies vary considerably, from 10 mg/kg to 5000 mg/kg feed in certain experimental formulations, depending on the type of product and study design. However, most studies report inclusion levels within the range of 100 to 400 mg/kg feed, administered over periods of 21 to 52 days. Curcumin is predominantly used in its standard form, but also as nanoformulations, which may influence dosage levels and biological efficacy. These results support the potential relevance of curcumin in nutritional strategies aimed at improving poultry health and efficiency under intensive production conditions [2,7]. Curcumin supplementation was associated with reduced oxidative stress and tissue lesions at both hepatic and ileal levels, contributing to the attenuation of mycotoxin-induced toxic effects and supporting its usefulness in their nutritional management [41]. Its veterinary relevance is further supported by studies based on experimental challenge models. In broiler chickens exposed to feed contaminated with aflatoxin B1, the effects of curcumin were evaluated in a 28-day study using four experimental groups: control, AFB1 at 1 mg/kg, CUR at 500 mg/kg and AFB1 + CUR [42]. Similarly, protective effects have been reported in broiler chickens exposed to low levels of aflatoxin B1, at 0.02 mg/kg feed, where the administration of 400 mg/kg dietary curcumin for 10 days was associated with reduced renal oxidative stress. In addition, curcumin supplementation in broiler chickens infected with *Eimeria tenella* improved the anticoccidial index and was associated with increased antioxidant capacity, reduced inflammatory response and improved intestinal barrier function. In this case, the birds received 200 mg/kg dietary curcumin in a 42-day feeding study [43]. Overall, these data indicate that curcumin may have a relevant role in poultry production not only in supporting performance but also as an adjuvant under conditions associated with enteric and toxicological stress.

### 5.2. Swine

In swine, the most relevant context for curcumin supplementation is the post-weaning period, which is frequently associated with oxidative stress, impairment of intestinal barrier function and reduced growth performance. In Wuzhishan piglets, dietary administration of curcumin, either individually or in combination with piperine, improved feed efficiency, reduced serum markers of intestinal permeability and increased antioxidant capacity, indicating a beneficial effect on intestinal integrity and redox balance [44]. In this context, in weaned Wuzhishan piglets aged 35 days, curcumin was administered in the diet at doses of 200 and 300 mg/kg, alone or in combination with piperine at 50 mg/kg, within an experimental design involving five groups. These observations are of veterinary interest, as intestinal dysfunction during the weaning period remains a major challenge in swine production.

Curcumin has also been evaluated in piglets with intrauterine growth restriction, where dietary supplementation increased hepatic antioxidant capacity and upregulated responses associated with the Nrf2 and Hmox1 pathways [45]. In this context, in weaned piglets with intrauterine growth restriction (IUGR), the effects of curcumin were evaluated in an experimental study with four groups: NBW (normal-birth-weight), NBW + curcumin, IUGR and IUGR + curcumin. Dietary supplementation was associated with improved antioxidant capacity and growth performance. In this context, curcumin may contribute to the nutritional support of piglets with increased metabolic vulnerability. Although the available studies are encouraging, the current evidence remains largely experimental, and validation under commercial production conditions is required [44,45].

### 5.3. Ruminants

In ruminants, curcumin has mainly been studied in relation to ruminal fermentation, microbial protein synthesis and systemic antioxidant status. In growing lambs, supplementation improved growth performance, ruminal fermentation parameters, ruminal microbial protein synthesis and certain indicators of serum antioxidant capacity [46]. In this context, in lambs aged approximately 120 days, curcumin was administered in the diet at doses of 300, 600 and 900 mg/kg, within a randomised experimental design involving four groups: control, 300 CUR, 600 CUR and 900 CUR. This was associated with improved ruminal fermentation, microbial protein synthesis and antioxidant capacity. At the same time, higher doses were associated with changes in specific ruminal microbial populations, suggesting the importance of optimising the level of administration in systems dependent on fibre digestion [46]. From a veterinary and nutritional perspective, these results indicate that curcumin may support ruminal function and oxidative balance, although its effects should be interpreted in the context of the complexity of the ruminal ecosystem.

In dairy cows, turmeric has more frequently been evaluated within multicomponent phytogenic formulations than as an isolated substance. In Jersey cows, the administration of a mixture of free and microencapsulated essential oils, turmeric and tannins was associated with improved productive efficiency and certain health status parameters [47]. In this type of approach, a phytobiotic additive containing cinnamon and oregano essential oils, turmeric extract and tannins was administered at a dose of 2 g/cow/day for 45 days, within an experimental design involving two groups: control and treatment. This was correlated with improvements in productive efficiency, milk composition, and antioxidant and immune status. Convergent results were also reported in lactating Jersey cows receiving combinations of phytogenic compounds that included turmeric extract, with beneficial effects observed on productive efficiency, milk composition, the ruminal environment and certain indicators of health status [48]. In addition, the administration of a complex phytoactive mixture over two lactation phases of 45 days each was associated with improved milk production and quality, ruminal environment and health status. These results are relevant for the veterinary management of herds and for nutritional support; however, the effects should be interpreted at the level of the entire phytogenic formulation and should not be attributed exclusively to curcumin.

### 5.4. Aquaculture

In aquaculture, curcumin is increasingly being investigated as a natural feed additive with the potential to improve growth, immune response and oxidative balance. In gilthead seabream (*Sparus aurata*), dietary administration of curcumin improved growth and haematobiochemical parameters and increased intestinal antibacterial capacity [8]. It was used in the diet at levels of 1.5 to 3% over a period of 150 days, within a controlled experimental design. In snakehead fish (*Channa argus*), curcumin supplementation improved growth performance and attenuated the inflammatory response following a lipopolysaccharide challenge test [49]. This was observed under conditions in which doses of 0, 100, 200, 400 and 800 mg/kg were administered for 8 weeks, within a five-group experimental design. In red tilapia (*Oreochromis* sp.), supplementation was associated with improved growth performance, feed utilisation efficiency, redox balance and certain immunological parameters, as well as favourable histological changes and increased expression of antioxidant genes in the liver [50]. Levels of 0.4, 0.6 and 0.8 g/kg diet were used over a period of 60 days, within a four-group design.

The hepatic relevance of curcumin is also highlighted under combined stress conditions. In grass carp (*Ctenopharyngodon idella*) exposed to ochratoxin A and hypoxia, supplementation with 400 mg/kg curcumin for 60 days, within a four-group design comprising control, OTA at 1.2 mg/kg, CUR and OTA + CUR, was associated with reduced liver lesions, oxidative stress and cellular apoptosis [51]. These findings are particularly relevant for fish farming systems, where nutritional, toxicological and environmental factors may act simultaneously and directly influence animal health and productivity. Overall, the evidence suggests that curcumin may have practical value as a supportive dietary component in aquaculture, particularly under intensive farming conditions.

### 5.5. Companion Animals

In companion animals, the most relevant clinical evidence regarding the use of curcumin currently concerns the management of osteoarthritis. In dogs, a randomised, double-blind, placebo-controlled study showed that a diet supplemented with curcuminoid extract, hydrolysed collagen and green tea extract may improve pain-related indicators, although the main objective parameters did not differ significantly between groups [5]. Administration of this mixture for 3 months was associated with reduced pain on manipulation and improvements in certain functional parameters, suggesting a potential clinical benefit, despite the moderate level of evidence. More recent data support the role of curcumin-containing nutraceuticals in canine joint disease. A supplement including Curcumin C3 Complex, glucosamine and chondroitin, administered for 30 days, was associated with reduced serum inflammatory markers, namely MMP-3 (matrix metalloproteinase-3) and TNF-α (tumour necrosis factor alpha), and pain relief [52], strengthening interest in the use of curcumin within multimodal nutritional strategies. In cats, a diet enriched with EPA (eicosapentaenoic acid), DHA (docosahexaenoic acid), turmeric extract and hydrolysed collagen demonstrated beneficial effects in naturally occurring osteoarthritis [53]. Administered over a period of 13 weeks, followed by a 4-week washout period, it was associated with improved mobility, reduced pain and improved joint function, with no adverse effects observed. Overall, these data indicate that turmeric-derived ingredients may represent useful adjuvant nutritional tools in companion animal medicine, particularly in chronic musculoskeletal disorders. However, the multicomponent nature of these formulations limits the possibility of attributing the observed effects exclusively to curcumin.

In addition to its effects in musculoskeletal disorders, curcumin has also been investigated for its anticancer potential in veterinary medicine. In dogs with spontaneously occurring tumours, a liposomal curcumin formulation, Lipocurc™, (SignPath Pharma Inc., Quakertown, PA, USA), demonstrated antiproliferative and antiangiogenic effects in vitro. In a pilot clinical study, it was administered intravenously at a dose of 10 mg/kg, as 8 h infusions repeated weekly for 4 weeks, and was associated with disease stabilisation in some animals, without significant tumour regression, suggesting a possible adjuvant role [54]. Experimental evidence, predominantly in vitro, supports these observations. Studies on canine cell lines have shown dose-dependent antiproliferative effects and induction of apoptosis, both in mammary tumours (2.5 to 200 µM, 24 to 72 h) and in urothelial carcinoma (12,000 to 18,000 ng/mL, 72 h), including in combination with chemotherapeutic agents, where synergistic effects were observed [55,56]. Experimental studies in other animals also indicate the ability of curcumin to inhibit tumour growth and modulate oxidative stress and signalling pathways involved in carcinogenesis; however, these data remain largely preclinical [57]. Nevertheless, clinical evidence remains limited, with most data originating from in vitro studies or pilot studies with small sample sizes. In addition, the low bioavailability of curcumin may limit its clinical efficacy, justifying the use of advanced formulations such as liposomal systems or nanoformulations [54,57]. Overall, curcumin shows promising potential as a complementary therapy in veterinary oncology, particularly in canine cancers. However, well-designed in vivo studies and large-scale controlled clinical trials are required to validate its efficacy and establish standardised therapeutic protocols [54,56,57].

The veterinary applications of *C. longa* and curcumin across different animal species are summarized in Table 2.

However, from a practical perspective, large-scale applicability in animal production also depends on economic aspects, including formulation costs and the cost–benefit ratio, particularly in comparison with conventional additives.

## 6. Bioavailability, Stability, Safety and Formulation Strategies for the Veterinary Use of *C. longa*-Derived Products

### 6.1. Why Bioavailability Remains a Major Limitation for Translation into Veterinary Medicine

Curcumin is characterised by low oral bioavailability, and this limitation may contribute to variability in systemic exposure and difficulties in reproducing systemic effects in clinical studies. Based on a single methodological analysis, it was reported that the systematic reviews evaluated in that study did not always adequately account for differences in bioavailability among curcumin formulations, for example, through stratification or sensitivity analyses. Therefore, this observation should be interpreted within the context of that specific study and may indicate a possible source of heterogeneity in the interpretation of clinical evidence [58]. A pharmacokinetic study reported that after oral administration, plasma concentrations of unconjugated curcumin remain very low, even at high doses and in formulations marketed as having enhanced absorption. In the same study, co-administration with piperine did not result in a measurable increase in systemic exposure to unconjugated curcumin under the evaluated conditions. These findings suggest that the so-called bioenhancer effect should not be assumed to be constant across contexts, and its extrapolation as a universal solution requires caution and empirical confirmation, particularly when the population, dose or target species changes [59].

It is crucial to emphasize that the limitations related to bioavailability and exposure variability do not concern isolated curcumin alone but may also affect the interpretation of results obtained with *C. longa*-based products, including standardized extracts or rhizome preparations, since the matrix and route of administration may influence the release and solubilisation of compounds in the gastrointestinal tract [58].

### 6.2. Chemical and Technological Stability, with Relevance for Feed Applications

Curcumin may undergo degradation under thermal treatment conditions, and the resulting degradation products may exhibit biological activity of their own, which may complicate the estimation of actual exposure and the “effective dose” in processed products. This observation has potential implications for applications in animal production, since feed processing steps such as conditioning and performance may involve high temperatures that could affect the stability of curcumin and the profile of compounds administered [60]. Microencapsulation is a technological strategy that has been investigated to protect curcumin and preserve its functional properties after moderate thermal stress [61]. In addition to temperature, storage stability is an important practical variable, and some nanoemulsion-based systems may improve stability over several weeks under storage conditions [62].

Consequently, in order to improve comparability among studies, it is important to report the characteristics of the products used, including aspects related to processing and storage, since these factors may influence chemical stability and biological exposure [58].

### 6.3. Strategies to Increase Bioaccessibility and Exposure

Formulations may increase oral exposure to curcuminoids by improving solubilisation and the processes that facilitate passage across the intestinal barrier, and this principle is supported by comparative absorption studies of different curcumin delivery systems. Several formulations have been evaluated, including a phytosomal formulation and a formulation associated with volatile oils from the rhizome, with differences being reported in the appearance of curcuminoids in plasma compared with unformulated curcumin, based on analyses performed after enzymatic deconjugation of the samples [63]. From a methodological perspective, the findings of this study should be interpreted as a context-specific observation rather than as a general conclusion. They suggest that the matrix and delivery system may modify internal exposure to curcuminoids and may therefore influence the magnitude of the observed effect. This aspect should be considered with caution when interpreting veterinary studies, particularly when comparing conventional curcumin preparations with formulations designed to enhance absorption, such as those containing piperine [58]. In the field of animal nutrition, curcumin nanoformulations are discussed as an emerging option for increasing bioavailability and achieving more consistent effects in monogastric animals, poultry and fish [64]. In aquaculture, effects of nanocurcumin on performance and selected health indicators have been reported in red tilapia, including in the context of challenge with *Aspergillus flavus* [65]. With regard to improving bioaccessibility, curcumin-piperine complexes incorporated into nanoemulsions stabilized with phospholipids and whey protein have been evaluated, with improved storage stability and increased bioaccessibility under simulated gastrointestinal digestion conditions being reported, depending on the formulation [62].

At the same time, controlled pharmacokinetic data suggest that piperine does not necessarily increase systemic exposure to unconjugated curcumin under the evaluated conditions and, therefore, presenting piperine as a universally valid solution requires caution and species-specific confirmation [59].

### 6.4. Safety and Toxicological Considerations in Target Species

Curcumin and preparations derived from *C. longa* are generally considered to have a favourable safety profile, with low toxicity reported in experimental and clinical studies [66,67]. Experimental studies indicate good tolerability at moderate levels of supplementation. Doses of approximately 200 mg/kg feed in broiler chickens, 200 mg/kg diet in growing pigs, 100 mg/kg diet in marine fish and 50 to 200 mg/kg diet for curcumin or nano-curcumin in tilapia have been reported without evidence of major clinical adverse effects under the experimental conditions evaluated [68,69,70,71]. The assessment carried out by the EFSA FEEDAP Panel indicates that preparations derived from turmeric are safe for target species when used in feed or drinking water at the proposed inclusion levels, generally expressed in mg/kg of feed or mg/L of water, depending on the type of preparation and species [72]. For example, extracts are considered safe at levels of approximately 15 mg/kg feed, supporting the controlled use of products derived from *C. longa* in animal nutrition. Data from applied poultry studies confirm these observations, with curcumin supplementation in the range of 100 to 200 mg/kg feed being associated with improved productive performance, antioxidant status and certain metabolic parameters, without the reporting of major clinical adverse effects under the experimental conditions evaluated [68,73]. These findings suggest that the use of curcumin at moderate doses is generally well tolerated.

However, safety may be influenced by factors such as dose, duration of administration, type of formulation and species-specific metabolism [66]. High levels or long-term administration may induce physiological or metabolic changes, although evidence regarding clinically relevant toxicity remains limited. Although adverse effects are considered rare at usual supplementation levels, some studies suggest possible effects at high doses; however, these observations are dose-dependent, influenced by experimental conditions and not consistent across studies. The interpretation of results is further complicated by the frequent use of multicomponent formulations, in which curcumin is combined with other bioactive compounds, making it difficult to attribute the observed effects exclusively to curcumin. In addition, strategies designed to increase bioavailability, such as nanoformulations or combinations with absorption-enhancing agents, may modify systemic exposure and, consequently, influence the safety profile, requiring careful toxicological evaluation. Overall, current evidence supports a favourable safety profile for products derived from *C. longa* in target species when used at appropriate doses. Nevertheless, further studies are required to define optimal dosage ranges, evaluate long-term safety and identify any species-specific adverse effects under real production conditions.

### 6.5. Food Safety and Consumer Health Considerations

From the perspective of food safety, the pharmacokinetic profile of curcumin is characterised by low oral bioavailability and rapid metabolism. These properties are reflected in reduced plasma levels of curcumin, even following oral administration at relatively high doses, indicating limited systemic exposure [66]. This reduced exposure may indirectly influence the potential transfer of compounds into edible tissues. Consequently, the accumulation of curcumin in meat, milk or eggs is likely to be limited, although direct evidence in food-producing animals remains insufficient. In this context, low bioavailability may be considered a favourable characteristic from a food safety perspective, as it could reduce the likelihood of the persistence of significant residues. However, this interpretation is based mainly on indirect pharmacokinetic data, in the absence of systematic studies specifically dedicated to residue assessment. This aspect becomes even more relevant in the context of the increasingly frequent use of phytogenic additives in the diets of animals intended for food production [66].

Curcumin is generally considered to have a favourable safety profile, with low toxicity reported in both experimental and clinical studies, and has been assessed as safe under established conditions of use. The FEEDAP assessment indicates that preparations derived from *C. longa* are safe for target species and do not pose a risk to consumers when used at the recommended levels. However, compliance with these limits is essential, as high intakes may exceed the values considered safe in risk assessments [72].

At present, studies specifically addressing residue kinetics, tissue distribution and long-term exposure through food products of animal origin are limited. In addition, variability in formulation, dose, duration of administration and species-specific metabolism may influence systemic exposure and the potential presence of metabolites in edible tissues, aspects that are consistent with general observations regarding the variability of systemic exposure to curcumin [66]. Therefore, although the current evidence suggests a low risk of accumulation and a favourable safety profile, definitive conclusions regarding consumer safety cannot yet be formulated. Further studies are needed to characterise residue levels, metabolic fate and possible regulatory thresholds in food-producing animals in order to support the safe and standardised use of products derived from *C. longa*. These aspects are particularly relevant in the context of the growing interest in the use of phytogenic additives in animal production systems intended for human consumption.

### 6.6. Recommendations for Reporting, Comparability and Translation

For comparability among studies, clear reporting of the type of curcumin-based product used and its chemical characterization, for example, curcuminoid content and degree of standardization, is essential, since differences among products are often insufficiently integrated into existing syntheses [58]. For advanced formulations, detailed characterization of the system, for example, particle size and stability, as well as parameters related to compound loading, is important for critical evaluation and comparability among studies, particularly when nano-systems are discussed as strategies to improve bioavailability [64]. For processed feeds, temperature conditions and processing time should be explicitly reported, since heat exposure may induce degradation and changes in the chemical profile, with a potential impact on biological activity [60]. When evaluating claims related to bioavailability, it is important to specify whether the reported values refer to unconjugated curcumin or its conjugates because this distinction may influence the interpretation of systemic exposure [59].

In addition, strategies aimed at increasing exposure should be discussed together with potential safety implications and interactions, since altered exposure may affect the risk-benefit profile, particularly in animals [73].

## 7. Methodological Limitations and Future Directions

The interpretation of results regarding the use of *C. longa* and curcumin in veterinary medicine is limited by the variability of the products used, including rhizome powder, standardised extracts, liposomal formulations, nanoformulations and multicomponent products, differences that influence the comparability and reproducibility of studies. The lack of standardisation represents a major issue, as variations in phytochemical composition, curcuminoid content, form of administration and dosage may affect the comparability of results across studies [57,66]. Future studies should clearly report the type of product used, the active substance content, the dose expressed both as total product and as active principle, the duration and route of administration, and the use of standardised control groups that are comparable across studies in order to allow a rigorous and reproducible evaluation of results [66,72]. Although advanced formulations, such as liposomal systems and nanoformulations, may improve systemic exposure, the available data do not include sufficient standardised direct comparisons between these forms and conventional formulations. In general, improved formulations appear to offer advantages in terms of bioavailability; however, these benefits are not always consistently reflected in biological outcomes, and their superiority over conventional forms remains dependent on the species and experimental context.

In addition, many veterinary studies use multicomponent products, which limits the ability to attribute the observed effects exclusively to curcumin or preparations derived from *C. longa* [47,53]. Furthermore, variations in formulation, including the use of liposomal systems or other modified forms, may influence the biological response and complicate the comparative interpretation of results [54]. This issue is particularly relevant in animal studies, where curcumin is frequently administered in combination with other bioactive substances, such as collagen, green tea, glucosamine or chondroitin [5,52]. In ruminants, the use of phytogenic mixtures that include turmeric extracts alongside essential oils or tannins has also been reported, which may complicate the interpretation of individual effects [47]. Therefore, future research should include experimental designs that allow a clear differentiation between the effects of curcumin and those of complex formulations, including through the use of appropriate comparative groups. For practical applicability, standardised protocols, the evaluation of dose–response relationships and the integration of pharmacokinetic data are required [66,72]. In addition, the assessment of the cost–benefit ratio under real production or clinical use conditions represents an important aspect that requires further investigation.

## 8. Conclusions

Curcumin and products derived from *C. longa* represent promising phytogenic resources for veterinary medicine and animal production due to their antioxidant, anti-inflammatory, antimicrobial and immunomodulatory properties. These properties have been demonstrated through beneficial effects on productive performance, oxidative status, intestinal health and immune response in multiple animal species, thereby constituting an important strength and supporting interest in their use as alternatives to conventional antimicrobials. However, their practical applicability is limited by several weaknesses, particularly low oral bioavailability, the lack of extract standardisation and the considerable variability in doses and experimental protocols, which make it difficult to compare results and formulate clear veterinary recommendations. At the same time, relevant opportunities exist in relation to the development of advanced formulations, such as nano- and liposomal systems, and the integration of curcumin into modern nutritional strategies aimed at supporting health and reducing antimicrobial use. Nevertheless, these prospects are accompanied by certain threats, including the costs associated with innovative formulations, the lack of large-scale clinical studies, regulatory difficulties and the risk of unjustified extrapolation of results between species. Overall, although curcumin shows considerable potential as a nutritional and therapeutic adjuvant, the validation of its clinical use requires well-designed in vivo studies, product standardisation, and the evaluation of dose–response relationships and the cost–benefit ratio under real production conditions.

## Figures and Tables

**Table 1:** Main biological and functional mechanisms associated with compounds from *C. longa*.

Activity	Main Compound/Fraction	Main Mechanism or Pathway	Key Targets/Markers	Functional Significance	Ref.
Antioxidant and cytoprotective activity	Curcumin	Modulation of the Keap1–Nrf2–ARE axis and induction of cytoprotective genes	Nrf2, ARE, HO-1, NQO1	Supports cellular antioxidant defense and protection against oxidative stress	[32,33]
Antioxidant and cytoprotective activity	Curcumin	Multi-target regulation of interconnected redox signaling networks	Nrf2/ARE and related redox pathways	Contributes to redox homeostasis and cellular adaptation to oxidative stress	[32,33]
Anti-inflammatory activity	*Curcuma* spp.; curcumin	Modulation of inflammatory signaling pathways and suppression of pro-inflammatory mediators	NF-κB, COX-2, iNOS, inflammatory cytokines and chemokines	Reduces inflammatory signaling and mediator production	[34]
Anti-inflammatory activity	Curcumin	Inhibition of NLRP3 inflammasome activation in PMA-differentiated THP-1 macrophages	NLRP3, caspase-1, IL-1β, TLR4, MyD88, NF-κB, P2X7R	Attenuates inflammatory responses and decreases IL-1β maturation and secretion	[35]
Antimicrobial activity	Curcumin	Anti-infective activity involving multiple microbial targets and interference with virulence-related processes	Biofilm formation, quorum sensing	Suggests antibacterial potential, including against hard-to-control bacterial phenotypes	[36]
Antimicrobial activity	Curcumin and derived formulations	Antimicrobial efficacy may vary according to formulation-dependent exposure	Solubility, aqueous stability, bioavailability	Product form and formulation may substantially influence reported outcomes	[37,38]
Immunomodulation	*Curcuma* spp.; bioactive compounds, especially from *C. longa*	Modulation of inflammatory mediators, cytokines and intracellular signaling	NF-κB, inflammatory cytokines	Supports the immunomodulatory potential of *Curcuma*-derived compounds	[39]
Immunomodulation	Crude extracts and *Curcuma*-derived products	Limited extract characterization complicates mechanistic interpretation	Poor chemical standardization	Reduces the ability to assign biological effects to specific metabolites	[39]
Contribution of the volatile fraction and distinction from curcuminoids	Essential oil and volatile fraction	Bioactivity associated with mechanisms only partly overlapping with those of curcuminoids	α-turmerone, ar-turmerone, β-turmerone	Highlights the need to distinguish volatile from non-volatile fractions	[18]
Contribution of the volatile fraction and distinction from curcuminoids	β-turmerone and dichloromethane fraction	Anti-inflammatory activity identified by activity-guided isolation	β-turmerone	Suggests a relevant contribution of the volatile fraction to anti-inflammatory activity	[40]
Contribution of the volatile fraction and distinction from curcuminoids	*C. longa*-derived products	Explicit reporting of product type is essential for interpretation	Essential oil, non-volatile fraction, curcuminoid-rich extract	Chemical profile and bioactivity depend on the analyzed fraction and extraction conditions	[15]

**Table 2:** Veterinary applications of *C. longa* and curcumin across animal species.

Species/Category	Experimental Model/Condition	Product and Dose	Duration of Administration	Main Reported Effects	Ref.
Broiler chickens	Dietary supplementation under normal production conditions	Curcumin, 10 to 5000 mg/kg feed; most commonly 100 to 400 mg/kg feed	21 to 52 days	Increased average daily gain, improved feed conversion, improved antioxidant status and intestinal morphology	[2,7]
Broiler chickens	Aflatoxin B1 challenge	AFB1, 1 mg/kg feed; curcumin, 500 mg/kg feed	28 days	Reduction of oxidative stress and hepatic and ileal lesions	[41]
Broiler chickens	Exposure to low levels of aflatoxin B1	AFB1, 0.02 mg/kg feed; curcumin, 400 mg/kg feed	10 days	Reduction of renal oxidative stress	[42]
Broiler chickens	Experimental infection with *Eimeria tenella*	Curcumin, 200 mg/kg feed	42 days	Improved anticoccidial index, increased antioxidant capacity, reduced inflammation and support of intestinal barrier function	[43]
Weaned Wuzhishan piglets	Post-weaning stress and impaired intestinal integrity	Curcumin, 200 or 300 mg/kg feed, alone or with piperine, 50 mg/kg feed	NR	Improved feed efficiency, reduced intestinal permeability markers and increased antioxidant capacity	[44]
Piglets with intrauterine growth restriction	Metabolic vulnerability and hepatic oxidative stress	Dietary curcumin	NR	Increased hepatic antioxidant capacity, upregulation of the Nrf2 and Hmox1 pathways and improved growth performance	[45]
Growing lambs	Evaluation of ruminal fermentation, microbial protein synthesis and antioxidant status	Curcumin, 300, 600 or 900 mg/kg diet	NR	Improved growth performance, ruminal fermentation, microbial protein synthesis and serum antioxidant capacity	[46]
Jersey cows	Phytobiotic additive with essential oils, turmeric extract and tannins	Phytogenic mixture, 2 g/cow/day	45 days	Improved productive efficiency, milk composition, antioxidant status and certain immunological indicators	[47]
Lactating Jersey cows	Multicomponent phytogenic formulations including turmeric extract	Complex phytoactive mixture	Two phases of 45 days each	Improved milk production and quality, ruminal environment and certain health status indicators	[48]
Gilthead seabream (*Sparus aurata*)	Dietary supplementation in aquaculture	Curcumin, 1.5 to 3% in the diet	150 days	Improved growth, haematobiochemical parameters and intestinal antibacterial capacity	[8]
Snakehead fish (*Channa argus*)	Inflammatory challenge with lipopolysaccharides	Curcumin, 0, 100, 200, 400 or 800 mg/kg diet	8 weeks	Improved growth performance and attenuation of the inflammatory response	[49]
Red tilapia (*Oreochromis* sp.)	Dietary supplementation for growth, immunity and antioxidant status	Curcumin, 0.4, 0.6 or 0.8 g/kg diet	60 days	Improved growth, feed efficiency, redox balance, immunological parameters and expression of hepatic antioxidant genes	[50]
Grass carp (*Ctenopharyngodon idella*)	Exposure to ochratoxin A and hypoxia	OTA, 1.2 mg/kg diet; curcumin, 400 mg/kg diet	60 days	Reduction of hepatic lesions, oxidative stress and cellular apoptosis	[51]
Dogs with osteoarthritis	Functional diet for the management of joint pain	Curcuminoid extract, hydrolysed collagen and green tea extract	3 months	Reduced pain on manipulation and improvement of certain functional parameters	[5]
Dogs with joint disorders	Multicomponent nutraceutical supplement	Curcumin C3 Complex, glucosamine and chondroitin	30 days	Reduction of serum inflammatory markers MMP-3 and TNF-α and pain relief	[52]
Cats with naturally occurring osteoarthritis	Functional diet for supporting joint mobility	EPA, DHA, turmeric extract and hydrolysed collagen	13 weeks, followed by a 4-week washout period	Improved mobility, reduced pain and improved joint function, with no adverse effects observed	[53]
Dogs with spontaneous tumours	Pilot clinical study with liposomal formulation	Lipocurc™, 10 mg/kg intravenously, 8 h infusion, weekly	4 weeks	Disease stabilisation in some animals, without significant tumour regression	[54]
Canine tumour cell lines	In vitro models of mammary tumours and urothelial carcinoma	Curcumin, 2.5 to 200 µM for 24 to 72 h; 12,000 to 18,000 ng/mL for 72 h	in vitro	Dose-dependent antiproliferative effects, induction of apoptosis and possible synergistic effects with chemotherapeutic agents	[55,56]
Experimental tumour models	Evaluation of preclinical anticancer effects	Curcumin and derived formulations	NR	Inhibition of tumour growth and modulation of oxidative stress and signalling pathways involved in carcinogenesis	[54,57]

Note: AFB1, aflatoxin B1; curcumin; DHA, docosahexaenoic acid; EPA, eicosapentaenoic acid; MMP-3, matrix metalloproteinase-3; NR, not reported in the source text; OTA, ochratoxin A; TNF-α, tumour necrosis factor alpha.

## Data Availability

No new data were created or analyzed in this study. Data sharing is not applicable to this article.

## Abbreviations

DNA	Deoxyribonucleic acid
CpDNA	Chloroplast DNA
HPLC	High-Performance Liquid Chromatography
HPLC-MS/MS	High-performance liquid chromatography–tandem mass spectrometry
Keap1	Kelch-like ECH-associated protein 1
Nrf2	Nuclear factor erythroid 2–related factor 2
ARE	Antioxidant Response Element
HO-1	Heme oxygenase-1
NQO1	Quinone oxidoreductase 1
NF-κB	Nuclear factor kappa B
COX	Cyclooxygenase
iNOS	Inducible Nitric Oxide Synthase
NLRP3	The NOD-like receptor (NLR) family, the pyrin domain containing 3
THP-1	Monocytic cell line
PMA	Phorbol 12-myristate 13-acetate
IL-1β	Interleukin-1 beta
TLR4	Toll-like receptor 4
MyD88	Myeloid Differentiation Primary Response 88
P2X7R	Purinergic 2X7 receptor
AFB1	Aflatoxin B1
CUR	Curcumin
IUGR	Intrauterine growth restriction
NBW	Normal-birth-weight
OTA	Ochratoxin A
MMP-3	Matrix metalloproteinase-3
TNF-α	Tumour necrosis factor alpha
EPA	Eicosapentaenoic acid
DHA	Docosahexaenoic acid
LPS	Lipopolysaccharide
